# Differences in gut microbial fructoselysine degradation activity between breast-fed and formula-fed infants

**DOI:** 10.1093/femsec/fiac145

**Published:** 2022-11-28

**Authors:** Katja C W van Dongen, Athanasia Ioannou, Sebastiaan Wesseling, Karsten Beekmann, Clara Belzer

**Affiliations:** Division of Toxicology, Wageningen University and Research, Stippeneng 4, 6708 WE, Wageningen, The Netherlands; Laboratory of Microbiology, Wageningen University and Research, Stippeneng 4, 6708 WE, Wageningen, The Netherlands; Division of Toxicology, Wageningen University and Research, Stippeneng 4, 6708 WE, Wageningen, The Netherlands; Wageningen Food Safety Research, Wageningen University and Research, Akkermaalsbos 2, 6708 WB, Wageningen, The Netherlands; Laboratory of Microbiology, Wageningen University and Research, Stippeneng 4, 6708 WE, Wageningen, The Netherlands

**Keywords:** adaptation, AGEs, blastp, *in vitro* model, infant formula, infant gut microbiota, metagenome-assembled genomes

## Abstract

The Amadori product fructoselysine is formed upon heating of food products and is abundantly present in infant formula while being almost absent in breast milk. The human gut microbiota can degrade fructoselysine for which interindividual differences have been described for adults. The aim of this study is to compare functional differences in microbial fructoselysine degradation between breast-fed and formula-fed infants, in view of their different diets and resulting different fructoselysine exposures. First, a publicly available metagenomic dataset with metagenome-assembled genomes (MAGs) from infant fecal samples was analyzed and showed that query genes involved in fructoselysine degradation (frlD/yhfQ) were abundantly present in multiple bacterial taxa in the fecal samples, with a higher prevalence in the formula-fed infants. Next, fecal samples collected from exclusively breast-fed and formula-fed infants were anaerobically incubated with fructoselysine. Both groups degraded fructoselysine, however the fructoselysine degradation activity was significantly higher by fecal samples from formula-fed infants. Overall, this study provides evidence that infant formula feeding, leading to increased dietary fructoselysine exposure, seems to result in an increased fructoselysine degradation activity in the gut microbiota of infants. This indicates that the infant gut microbiota adapts towards dietary fructoselysine exposure.

## Teaser

Different dietary fructoselysine exposure affected bacterial composition as well as its metabolic capacity of infant gut microbiota.

## Abbreviations

AGE: advanced glycation end product
ASV: amplicon sequence variant
BF: breast-fed
CCA: canonical correspondence analysis
CE: collision energy
EDI: estimated daily intake
FF: formula-fed
LEfSe: linear discriminant analysis (LDA) effect size
LC: liquid chromatography
MAGs: metagenome-assembled genomes
MRM: multiple reaction monitoring
MS: mass spectrometry
PBS: phosphate-buffered saline

## Introduction

During the heating of food products, glycation products can be formed. These include the Amadori product fructoselysine, a precursor for the advanced glycation end product (AGE) carboxymethyllysine, which is produced via the non-enzymatic Maillard reaction from glucose and lysine residues during heating (Hodge 1953; Yaylayan et al. 1994). Depending on the nature of the lysine residue, free, peptide-bound, and/or protein-bound fructoselysine can be formed. During the production process of infant formula, extensive heating is applied to ensure microbial safety by which also high levels of fructoselysine are formed (Fenaille et al. 2006, Penndorf et al. 2007, Akıllıoğlu and Lund 2022). In human breast milk, in contrast, fructoselysine is (almost) absent with ∼239-fold lower levels compared to infant formula (Martysiak-Zurowska and Stolyhwo 2007). The question has been raised whether the fructoselysine degradation activity of infant gut microbiota adapts to these different exposure levels of fructoselysine due to the type of nutrition. It is known, for example, that the gut microbiota of formula-fed (FF) infants is distinct from that of breast-fed (BF) infants both with respect to its composition as well as its metabolic capacity (Wopereis et al. 2014, Bäckhed et al. 2015, Ma et al. 2020, Casaburi et al. 2021). In line with these observations, the aim of this study was to explore the ability of the infant gut microbiota to adapt towards different diets (BF or FF) with respect to their different levels of dietary derived fructoselysine.

**Figure 1.**
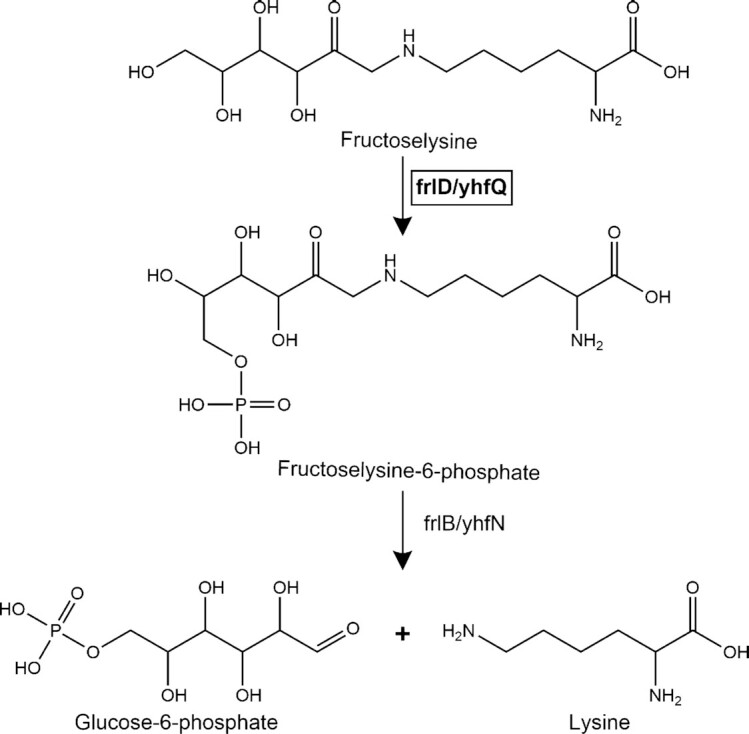
Pathway of fructoselysine degradation with identified genes involved. frlD/yhfQ encodes for fructoselysine kinase while frlB/yhfN encodes for fructosamine-6-phosphate deglycase.

Upon ingestion, fructoselysine can reach the colon and interact with gut microbiota (Erbersdobler and Faist 2001, Lee and Erbersdobler 2005), resulting in its degradation as shown by *in vitro* fermentations with human fecal slurries (Hellwig et al. 2015, van Dongen et al. 2021b) and with single bacterial strains (Wiame et al. 2002, 2004; Bui et al. 2015). In adults interindividual differences in microbial fructoselysine degradation have been reported (van Dongen et al. 2021b). An identified key enzyme in bacterial fructoselysine degradation is fructoselysine 6-kinase (encoded by frlD/yhfQ) which catalyzes ATP-dependent phosphorylation of fructoselysine into fructoselysine 6-phosphate, which can subsequently be converted into lysine and glucose 6-phosphate (Fig. 1) (Wiame et al. 2002, 2004; Bui et al. 2015). The genes coding for the kinase (frlD/yhfQ) were identified in multiple microbes (i.e. *Bacillus subtilis, Escherichia coli, Intestinimonas butyriciproducens* (Wiame et al. 2002, 2004; Bui et al. 2015)) and seems to be essential for bacterial fructoselysine degradation (Graf von Armansperg et al. 2020).

In FF infants aged 3 months, fecal fructoselysine excretion was reported to be 15.7 µmol/g feces while being absent in similar-aged BF infants (Sillner et al. 2019). The latter can be explained due to the absence of fructoselysine in breast milk, while the reported levels of fructoselysine in the feces of FF infants indicate that not all dietary exposed fructoselysine was absorbed or degraded by the gut microbiota. The composition of breast milk differs from infant formula in several ways (Su et al. 2017), including differences in fructoselysine levels. The gut microbiota can adapt towards dietary exposure, and in this study, it was investigated whether the ability of the infant gut microbiota to degrade fructoselysine also adapts towards different dietary fructoselysine exposure levels as part of different diets. To this end, a comparison in metagenomic differences and similarities of fructoselysine degradation genes between BF and FF infants was made with a publicly available metagenomic dataset (Bäckhed et al. 2015, Nayfach et al. 2019). In addition, BF and FF infant fecal samples were collected and compared for bacterial composition and *in vitro* metabolic activity to degrade fructoselysine. Fructoselysine levels were quantified in the infant formula of the FF infants to explore if differences in the consequent dietary fructoselysine exposure correlated with individuals’ fructoselysine degradation activities.

## Materials and methods

### Chemicals and reagents

Fructoselysine was purchased from Carbosynth Limited (Berkshire, UK). Glycerol, acetone, leucine aminopeptidase, pepsin, prolidase, pronase E and Tris were purchased from Sigma-Aldrich (Zwijndrecht, The Netherlands). *n*-Hexane was from VWR international (Amsterdam, The Netherlands). Formic acid (99%–100%, analytical grade) was purchased from Merck (Darmstadt, Germany). Phosphate-buffered saline (PBS) was obtained from Gibco (Paisley, UK). Methanol and acetonitrile (ACN), both in UPLC/MS grade were purchased from BioSolve BV (Valkenswaard, The Netherlands).

### Metagenomic data set analysis

Metagenome-assembled genomes (MAGs) (Nayfach et al. 2019) were filtered for the infant sequences of a publicly available data set (Bäckhed et al. 2015). The latter include metagenomic data from infants that are a few days, four months and twelve months old from various feeding backgrounds and birth modes. The nucleotide sequences were translated into amino acid sequences also based on protein-coding gene prediction, using ‘Prodigal’ (Hyatt et al. 2010) (version 2.6.3) in normal mode using default parameters. The MAGs were concatenated and transformed into a database with the use of the ‘makeblastdb’ command of the ‘blast’ (Altschul et al. 1990) (version 2.11.0) module. ‘Blastp’ was performed for the sequences presented in Table S1 against the created MAGs database. The taxonomy of the MAGs was based on the GTDB database (Parks et al. 2021). Hits were filtered for e-value < 1E-15, which resulted in no removal of hits. No other filtering was performed on the hits meaning that the hits are not necessarily functional proteins.

The aligned hits of ‘blastp’ were retrieved in FASTA files using the ‘seqinr’ package in R (version 4.0.2). Sequences were parsed for unique sequences per sequence, query gene and species name to avoid repetition in results. Alignment of amino acid sequences was performed with the build-in MUSCLE tool of MEGA (version 7.0.26) with default parameters. Aligned sequences were trimmed to equal lengths to avoid false divergence due to unequal lengths. The phylogenetic trees were created with the iqtree module (version 1.6.12). The Maximum-Likelihood model was used with the LG matrix (Le and Gascuel 2008) for 2 classes, empirical amino acid frequencies and FreeRates heterogeneity. The phylogenetic trees were further annotated with the iTOL software (version 6.3.2) (Letunic and Bork 2021). Prevalence of the query genes per feeding mode at 4 months were calculated by dividing the number of subjects per species, query and feeding mode by the total number of subjects per feeding mode multiplied by 100. Subjects with a feeding mode not assessed at 4 months were removed for this purpose. Additionally, the mean percentage identity of each hit per query and species found in each subject of 4 months of age was determined.

### Collection and processing of infant fecal samples

Infant fecal samples were collected and stored individually and assigned a random number. Parents from the infants granted informed consent before participation in this study. The study design was assessed by the Medical Ethical Committee of Wageningen University and judged not to fall under the Dutch ‘Medical Research Involving Human Subjects Act’. Fecal samples were collected from infants aged between 1 and 6 months who were either exclusively BF or FF (prior to introduction of solid foods), who did not receive antibiotics (when applicable up to 3 months prior to donation) and were born via vaginal childbirth. Fecal samples were scooped from the diaper into a sterile 50 ml filter top tube, which was directly stored in a BD GasPak^TM^ EZ Anaerobe gas generating pouch system with indicator (BD, Maryland, USA) and transferred to the fridge (± 4 °C). In addition, for the FF infants a scoop of formula powder was collected. Within 24 h, the fecal sample was further processed to fecal slurry under anaerobic conditions and stored at −80 °C after a 4x dilution (w/v) in anaerobic storage buffer (10% (v/v) glycerol in PBS; 0.25 g feces/mL) as described before (van Dongen et al. 2021b).

### In vitro anaerobic incubations with fecal slurries

Anaerobic incubations with the collected fecal slurries –individual or pooled– with fructoselysine were performed as previously described(van Dongen et al. 2021b). In short, two pools were created, containing equal amounts of either all FF infant fecal slurries or either all BF infant fecal slurries. 5% (v/v) Fecal slurry (final concentration of 0.0125 g feces/mL) from both pools were incubated in PBS for 0, 0.5, 1, 1.5, 2, 3, and 4 h with fructoselysine at 430 µM under anaerobic conditions. These experimental conditions were based on previous research using adult human fecal slurries (van Dongen et al. 2021b) and taking into account background fructoselysine concentrations in the pooled fecal samples of the FF infants so that the final concentration of fructoselysine was similar for the incubations with FF and BF samples.

Based on the pooled and previous results (van Dongen et al. 2021b), 5% (v/v) of infant fecal slurry (final concentration of 0.0125 g feces/mL) was found appropriate to study interindividual differences with all individual fecal slurries. To this end, background fructoselysine concentrations were quantified in these individual fecal slurries by LC-MS/MS as was done for the pool. The fecal slurries were subsequently incubated with a final concentration of 430 µM fructoselysine for 0, 2, 3, and 4 h. After the desired incubation time, reactions were terminated by addition of ice-cold ACN (1:1) and the samples were stored on ice for at least 15 min. All handlings were performed inside an anaerobic chamber (85% N_2_, 10% CO_2_ and 5% H_2_, Bactron EZ anaerobic chamber). Samples were centrifuged at 15 000 × *g* for 15 min at 4°C, and fructoselysine concentrations in the supernatants were analyzed by LC-MS/MS. All anaerobic incubations were performed in technical duplicates and were repeated at least three times.

### Sample preparation of collected infant formula samples

Both protein-bound and free fructoselysine were quantified in the collected infant formula samples based on the described procedure of Hegele *et al*. (2008).

Total fructoselysine levels were quantified upon digestion. After weighing 1 g of milk powder, 10 mL of 0.02 N HCl was added, and the milk suspension was weighed again. After mixing the suspension repeatedly for at least 15 min at room temperature, 50 µL of the milk suspension was transferred to an Eppendorf cup containing 950 µL 0.02 N HCl. The remaining milk suspension was reserved for protein determination, using the Pierce^TM^ BCA Protein Assay kit (Thermo Fisher Scientific, Illinois, USA), according to manufacturer's instructions.

To the milk suspension 18 µL of pepsin was added (1 mg/mL in 0.02 N HCl) and the sample was subsequently incubated for 1 h at 37°C. This was repeated, and followed by an addition of 250 µL 2 M Tris (pH 8.2) and 15 µL pronase E (1 mg/mL in 2 M Tris (pH 8.2)) after which the sample was incubated for 1 h at 37°C. Again, 15 µL pronase E solution was added followed by incubation for 1 h at 37°C. Subsequently, 20 µL leucine aminopeptidase (7 U/mL in H_2_O) and 10 µL prolidase (105 U/mL in 2 M Tris (pH 8.2)) were added and the sample was incubated for 24 h at 37°C. In the final step 15 µL pronase E was added and the incubation was continued for another 1 h at 37 °C. The samples were stored at −20°C until sample preparation for the LC-MS/MS. After thawing, the samples were centrifugated (15 000 × *g* for 30 min) and 500 µL of the supernatant was further prepared for LC-MS/MS as described below. An additional aliquot of each sample was spiked with fructoselysine for peak identification in the LC-MS/MS measurement.

Infant formula was prepared for free fructoselysine quantification as follows. In short, 1 gram of milk powder was weighed in a Greiner tube and 10 ml of nanopure H_2_O was added to the powder and the milk solution was weighed again. After mixing the suspension repeatedly during at least 15 min at room temperature, 1 ml of milk suspension was transferred to another 15 ml Greiner tube and weighed again. 10 ml of ice-cold acetone/MeOH (1:1, v/v) was added, after which the sample was vortexed and stored at −20°C for 1 h. After thawing, the sample was centrifugated (2000 × *g*, 5 min) and the supernatant was transferred to a glass tube. The supernatant was evaporated slowly under a flow of nitrogen gas while the tube was put in a heat block set at lukewarm. The dry remnant was resuspended in 1.4 ml nanopure H_2_O followed by adding 1.4 ml *n*-hexane. After vortexing the mixture thoroughly, it was kept at room temperature for 1 h until the separation between the two phases was sharp and clear, upon which the hexane fraction was removed. Then 500 µL of the water fraction was aliquoted and stored at −20 °C until sample preparation for LC-MS/MS carried out as described hereafter. An additional aliquot of each sample was spiked with fructoselysine for peak identification in the LC-MS/MS measurement.

Prior to LC-MS/MS measurement, samples were filtered. To this end, Nanosep 3 K Omega Filters of Pall Corporation (VWR international) were washed 5 times with nanopure H_2_O combined with a centrifugation step (12 000 × *g*, 2 min) before filtering the samples. Nanopure H_2_O was removed from the filter and the filter was transferred to a clean Eppendorf tube and loaded with 200 µL sample. After repeated centrifugation at 12 000 × *g* for 1 min at least 100 µL filtered sample was collected. The digestion filtrates were 25 times diluted in nanopure H_2_O and the free analyte filtrates were undiluted. All filtrates were subsequently analyzed by LC-MS/MS. Preparation and measurement of the infant formula samples was repeated three times.

### LC-MS/MS method and data processing to quantify fructoselysine in infant fecal slurries and infant formula samples

Fructoselysine concentrations were quantified using a Shimadzu Nexera XR LC-20AD SR UPLC system coupled to a Shimadzu LCMS-8040 triple quadrupole MS (Kyoto, Japan) for the anaerobic incubation samples, or coupled to a Shimadzu LCMS-8045 triple quadrupole MS (Kyoto, Japan) for the infant formula samples. The MS coupled to an ESI source was used for MS/MS identification, using positive ionization for multiple reaction monitoring (MRM), as previously described (van Dongen et al. 2021b). About 1 µL of either a milk or fecal incubation sample was injected onto a Phenomenex Polar-RP Synergi column (30 × 2 mm, 2.5 µm) at 40°C. The mobile phase consisted of a gradient made from solvent A (i.e. ultrapure H_2_O with 0.1% formic acid (v/v)) and solvent B (i.e. ACN with 0.1% formic acid (v/v)) at a flow rate of 0.3 ml/min. The gradient started with 95% ACN for 2.5 min, to reach 0% ACN at 4 min, and was subsequently kept at 0% ACN until 6 min, followed by a shift to 100% ACN from 6 to 7.8 min returning to 95% ACN at 8.1 min and kept at these initial conditions up to 14 min. Fructoselysine eluted at 5.6 min. The precursor to product transition *m/z* 309.2 > 84.2 (collision energy (CE) = −31 V) was used for quantification, while the transitions *m/z* 309.2 > 291.1 (CE = − 11 V), *m/z* 309.2 > 273.1 (CE = − 15 V) and *m/z* 309.2 > 225.2 (CE = − 17 V) were used as reference ions. Calibration curves of pure fructoselysine were used for quantification of fructoselysine present in the samples. Peak areas were integrated using LabSolutions software (Shimadzu).

The amount of degraded fructoselysine during the anaerobic incubations was expressed in µmol degraded/g feces and also in µmol degraded/1 × 10^11^ bacterial cells, or additionally relative to the duration of incubation, unless stated otherwise. Regarding the infant formula samples, quantified free fructoselysine was subtracted from the total fructoselysine quantified in the infant formula upon digestion to quantify the protein-bound fructoselysine levels. Protein-bound and free fructoselysine levels in infant formula were expressed per mg protein in the infant formula. Data of three repeated experiments were averaged and standard deviations were calculated with GraphPad Prism 5.0. Outliers were identified per feeding mode and time point using IBM SPSS Statistics version 25 using a multiplier of 3.0. When applicable, results were considered to be statistically significant when *P*-values were <0.05.

### DNA isolation, 16S rRNA amplicon sequencing, qPCR, and data processing

DNA was isolated from the fecal samples with a bead-beating procedure combined with the customized MaxWell® 16 Tissue LEV Total RNA Purification Kit (XAS1220; Promega Biotech AB, Stockholm, Sweden). The V4 region (515-F; 806-R) (Apprill et al. 2015, Parada, Needham and Fuhrman 2016) of the 16S ribosomal RNA (rRNA) gene was amplified by triplicate PCR reactions combined with a library approach as described before (van Dongen et al. 2021b). PCR products were purified, pooled and sequenced (Illumina NovaSeq 6000, paired-end; Eurofins Genomics Europe Sequencing GmbH, Konstanz, Germany).

With quantitative PCR (qPCR) reactions, the total bacterial load in each individual fecal slurry was quantified. Triplicate qPCR reactions were performed based on a previously described method (Wilms et al. 2021), where 1 µL DNA isolate (1 ng/µL) was mixed with 9 µL reaction mixture (containing 62.5% iQ SYBR Green Supermix, 2.5% forward primer (10 µM), 2.5% reverse primer (10 µM) and 32.5% nuclease free water). The total 16S rRNA gene was amplified with the following primer set: 1369-F (5′-CGG TGA ATA CGT TCY CGG-3′) and 1492-R (5′-GGW TAC CTT GTT ACG ACT T-3′). A standard curve was made with purified DNA isolate from *Escherichia coli* for quantification. The amplification program started at 95°C for 10 min, followed by 40 cycles of denaturing at 95°C for 15 sec, annealing at 60°C for 30 sec and elongation at 72 °C for 15 sec, to end with a melt curve from 60°C to 95°C, using a CFX-384 Touch Real-Time PCR detection system (Bio-Rad, California, USA). The CFX manager was used for data analysis. Quantified copy numbers of total 16S rRNA genes/g fecal sample were divided by the average 16S rRNA genes per bacterium (i.e. 4.2 (Větrovský and Baldrian 2013)) and further transformed to total bacterial load/g fecal sample. It is important to note that the dry weight of the fecal samples was not determined and thus differences in water content might have affected these results.

Sequences of the 16S rRNA gene were further processed with the NG-Tax 2.0 pipeline with default settings (Poncheewin et al. 2020), generating *de novo* exact match sequence clusters (ASVs; amplicon sequence variants). Taxonomy was assigned with the SILVA 16S rRNA gene reference database release 132 (Quast et al. 2013). Data were further analyzed with R (version 4.0.2). The ASV table was combined with the phylogenetic tree and metadata using the Phyloseq package (Mcmurdie and Holmes 2013) (version 1.34.0). A taxon present in at least one of the samples with a relative abundance >0.1% were included for further analyses, unless mentioned otherwise. By multiplying the relative abundance of a taxa within one sample with the corresponding total bacterial load as quantified by qPCR, absolute abundance data were created. This has been done as an approach to perform quantitative microbiome profiling as has been proposed in literature (Jian et al. 2020). Relative and absolute abundance plots of the top taxa were created with the Microbiome package (Lahti and Shetty 2017) (version 1.12.0). Bray-Curtis beta diversity was assessed with the Phyloseq package. Canonical correspondence analysis (CCA) was performed with the Vegan package (Oksanen et al. 2019) with feeding mode and age used as constraining factors. Spearman's rank correlations of fructoselysine degradation with microbial taxa present at a relative abundance of >0.1% in at least one of the samples and glomerated at genus and phylum level were made. *P* values were adjusted for multiple testing with the Benjamini & Hochberg false discovery rate using the Microbiome package (Lahti and Shetty 2017). The web-based tool to perform linear discriminant analysis (LDA) effect size (LEfSe) analysis (Segata et al. 2011) was used to identify differential abundant taxa as previously described (van Dongen et al. 2021b). Results were found to be statistically significant when *P*-values were <0.05.

## Results

### Metagenomic differences and similarities of fructoselysine degradation genes between fecal samples from BF and FF infants

To assess if fecal samples from BF and FF infants differ in their metabolic potential to degrade fructoselysine, a metagenomic dataset analysis was done with the metagenomes of a publicly available dataset (Bäckhed et al. 2015, Nayfach et al. 2019). For this purpose, the main focus was on the frlD/yhfQ genes (Fig. 1), which were shown to be crucial for fructoselysine degradation, which is catalyzed by the corresponding enzyme (Graf von Armansperg et al. 2020).

The MAGs of fecal samples from BF and FF infants included in the dataset showed multiple hits with the query genes (frlD/yhfQ) and were grouped on phylogenetic basis to retrieve insight in which bacterial taxa might harbor these genes and could potentially be involved in fructoselysine degradation (iTol tree Fig. S1). Especially a great diversity of taxa of the phylum Firmicutes appeared to be relevant, and to a lesser extent Actinobacteria, while only a few taxa of the phyla Proteobacteria and Bacteroidetes appeared to be potentially involved in fructoselysine degradation. For some genera a diversity of species belonging to the same overarching genus appeared to be potentially involved, as was the case for e.g. *Clostridium spp., Blautia spp., Collinsella spp*., and others. The genes involved in the second degradation step as shown in Fig. 1 (frlB/yhfN) were also redundant in multiple species part of different overarching genera and phyla as shown in the phylogenetic tree (iTol tree Fig. S2).

**Figure 2.**
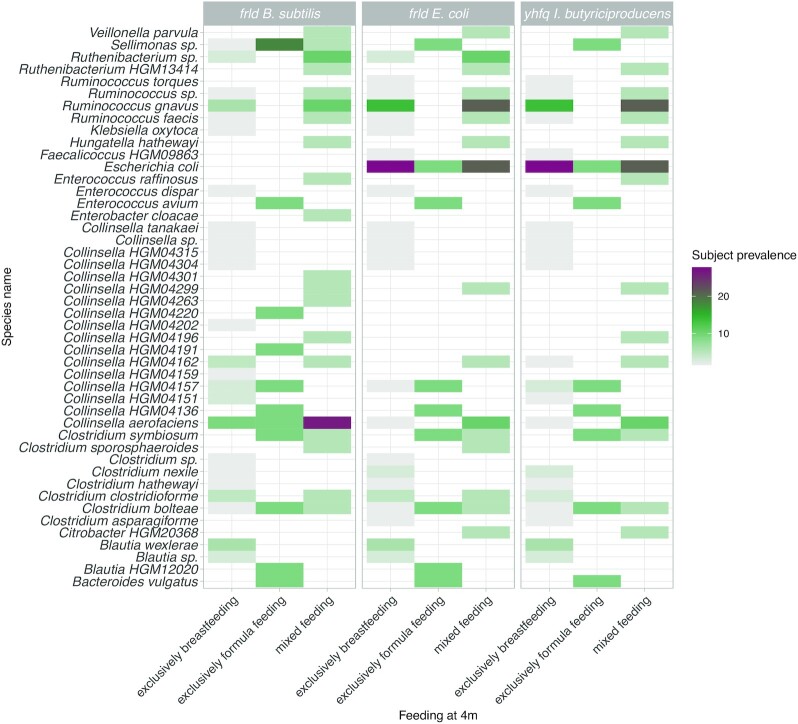
Heatmap of hits of the query genes against the included metagenome-assembled genomes (MAGs), for infants aged 4 months. Subject prevalence indicates the percentage of subjects that have a hit of each query per bacterial species (y-axis) and feeding mode of infants (x-axis). The taxa identity mentioned in the figure was the lowest identified taxonomic hierarchical level possible.

When only including the data of the BF, FF and mixed-fed 4 months old infants of the data set (Bäckhed et al. 2015), analysis revealed that the queries of frlD/yhfQ genes were present in multiple bacterial species and redundant in all diet groups. However, subject prevalence data (i.e. the number of subjects per feeding mode having a hit of the query genes per bacterial species) revealed that the query genes were in general present in a larger proportion of the microbiota of subjects in the FF infants, followed by those from the mixed feeding infants while they were lowest for the microbiota derived from the BF infants (Fig. 2; the mean percentage identity of each hit per query and species per subject can be found in Fig. S3).

### Bacterial composition of collected fecal samples from BF and FF infants

The individual fecal samples collected in the present study were analyzed for their bacterial composition by 16S rRNA amplicon analysis. In total 20 infant fecal samples were collected consisting of 10 exclusively BF infants (aged between 8 and 21 weeks, average 16 weeks) and 10 exclusively FF infants (aged between 7 and 21 weeks, average 13 weeks). The individuals of the two feeding modes did not result in two distinct clusters based on Bray–Curtis beta diversity dissimilarities (Fig. S4), indicating that the overall fecal bacterial composition did not differ between the two groups (FF and BF infants). To see whether this was the case for specific bacterial taxa, bacterial composition data at both phylum and genus level were averaged per feeding mode and shown in Table 1 (for bacterial composition plots per individual fecal sample in absolute abundances and relative abundances see Figs S5 and S6, respectively). This revealed that for most individuals, especially the genus *Bifidobacterium* was present in the individual fecal samples, which belongs to the phylum Actinobacteria, while for two individuals this genus was absent so these are considered as possible outliers (Individual 15 and 19). LEfSe analysis was performed to indicate differential abundant taxa between the two feeding modes, but no taxa were shown to be significantly different between the two groups with a LDA effect size > 2.0 (data not shown). This was confirmed by CCA analysis (canonical correspondence analysis) where age and feeding mode both did significantly explain some variation in the microbiota composition (adjusted *P-*values <0.05) but only for 8.7% and 6.8%, respectively (Fig. S7).

**Table 1.** Absolute abundance of microbial taxa, assessed with 16S rRNA amplicon sequencing and qPCR, present in fecal samples of either exclusively breast-fed or formula-fed infants. The top 10 taxa present at phylum and genus level are shown. Data represent averages ± SD per feeding mode, with in each group 10 individual infant fecal samples collected. The number of fecal samples that contained the bacterial taxa is indicated as well.

	Fecal samples from breast-fed infants		Fecal samples from formula-fed infants	
Bacterial taxa	Average ± SD	Present in number of samples	Average ± SD	Present in number of samples
***Phylum level***				
Actinobacteria	3.5 × 10^10^ ± 3.8 × 10^10^	9	3.7 × 10^10^ ± 2.5 × 10^10^	10
Firmicutes	1.6 × 10^10^ ± 3.2 × 10^10^	10	6.6 × 10^9^ ± 6.4 × 10^9^	10
Proteobacteria	1.1 × 10^10^ ± 1.4 × 10^10^	10	2.5 × 10^9^ ± 2.3 × 10^9^	10
Bacteroidetes	8.8 × 10^9^ ± 1.7 × 10^10^	5	2.9 × 10^9^ ± 3.3 × 10^9^	8
Verrucomicrobia	-	0	1.7 × 10^9^ ± 3.9 × 10^9^	2
***Genus level***				
*Bifidobacterium*	2.6 × 10^10^ ± 2.6 × 10^10^	8	3.5 × 10^10^ ± 2.3 × 10^10^	10
*Unclassified genus*	1.1 × 10^10^ ± 1.4 × 10^10^	10	2.2 × 10^9^ ± 2.3 × 10^9^	10
*Clostridium_sensu_stricto_1*	1.1 × 10^10^ ± 2.8 × 10^10^	4	2.3 × 10^8^ ± 4.5 × 10^8^	4
*Bacteroides*	7.2 × 10^9^ ± 1.7 × 10^10^	4	2.2 × 10^9^ ± 2.7 × 10^9^	7
*Actinomyces*	6.2 × 10^9^ ± 1.8 × 10^10^	4	2.2 × 10^7^ ± 3.6 × 10^7^	3
*Lactobacillaceae*	1.6 × 10^9^ ± 3.4 × 10^9^	5	1.3 × 10^9^ ± 3.4 × 10^9^	6
*Collinsella*	1.7 × 10^8^ ± 4.2 × 10^8^	2	2.0 × 10^9^ ± 3.4 × 10^9^	5
*Veillonella*	4.8 × 10^8^ ± 5.3 × 10^8^	7	1.5 × 10^9^ ± 1.8 × 10^9^	9
*Libanicoccus*	1.8 × 10^9^ ± 5.5 × 10^9^	1	-	0
*Akkermansia*	-	0	1.7 × 10^9^ ± 3.9 × 10^9^	2
*Other*	4.4 × 10^9^ ± 6.5 × 10^9^	9	4.6 × 10^9^ ± 5.6 × 10^9^	10

### Differences in fructoselysine degradation activities between BF and FF infant fecal samples *in vitro*

**Figure 3.**
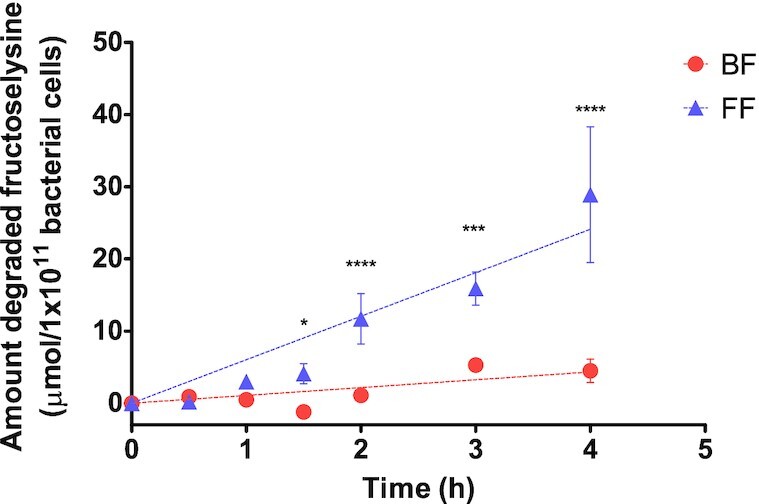
Amount of degraded fructoselysine upon anaerobic incubation of fructoselysine (in a final substrate concentration of 430 µM at t = 0 h) with pooled fecal slurries (final fecal concentration 0.0125 g/mL) of infants exclusively breast-fed (BF) or formula-fed (FF), containing 10 infant fecal samples per feeding mode per pool. Data points represent the average ± SD of three repeated experiments. Differences between the BF and FF results were assessed for statistical significance per time point by a 2-way ANOVA followed by Bonferroni post-hoc test: * *P*-value <0.05; ** *P*-value < 0.01; *** *P*-value < 0.001; ^****^P-value < 0.0001.

As the metagenomic dataset analysis revealed that fructoselysine degradation query genes were more prevalent in FF infants, it was investigated whether *in vitro* fructoselysine degradation activities by the collected fecal samples were also higher for the FF infants compared to the BF infants. First, pooled fecal slurries of both BF and FF infants were incubated anaerobically with fructoselysine up to four hours. The amount of fructoselysine degraded was corrected for the total bacterial cell load per gram feces as quantified by qPCR (for individuals’ total bacterial load see Fig. S8). Both groups were able to degrade fructoselysine, but the fructoselysine degradation was significantly and on average about 6-fold higher in FF infants compared to the BF infants from 1.5 h incubation onwards (*P*-value < 0.05) (degradation expressed per 1 × 10^11^ bacterial cells see Fig. 3; degradation expressed per gram feces see Fig. S9). For the first two hours of anaerobic incubation both groups seemed to have a lag phase. The largest absolute difference in fructoselysine degradation between FF and BF infants was observed at t = 4 h and amounted to 24.4 µmol/1 × 10^11^ bacterial cells.

**Figure 4.**
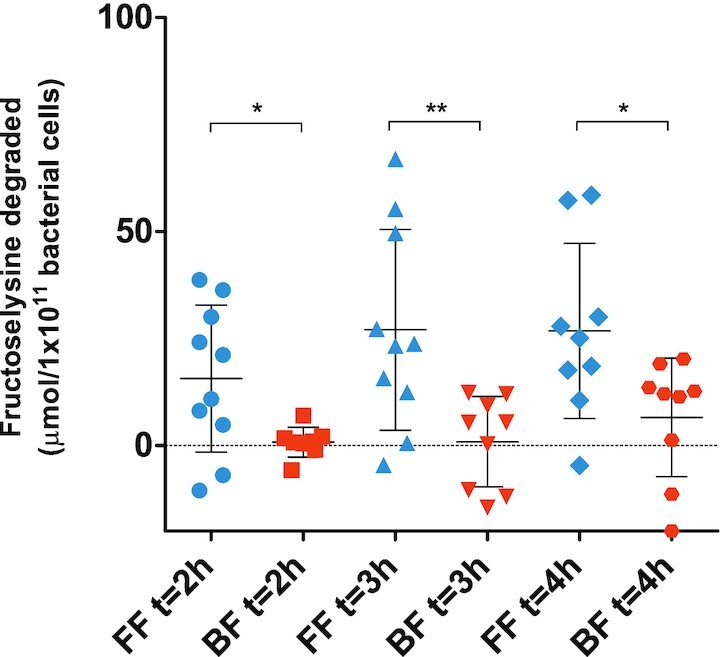
Amount of degraded fructoselysine by individual fecal samples from exclusively breast-fed (BF) or formula-fed (FF) infants quantified at each anaerobic incubation time point (i.e. 2, 3, or 4 h). Scatter dots indicate average values of three independent experiments for each individual fecal sample. Center bars indicate average values while whiskers indicate the standard deviation. Whether the values of the two feeding modes were significantly different for each respective incubation time was evaluated with an unpaired t-test: * *P*-value < 0.05; ** *P*-value < 0.01. Identified outliers were excluded.

To assess whether interindividual differences in the fructoselysine degradation capacity of the BF and FF infant fecal samples exist, all infant fecal samples collected were incubated individually under the same experimental conditions for 2, 3, and 4 h. Amounts of degraded fructoselysine were calculated per individual (see Fig. S10). Based on the feeding mode the amounts of degraded fructoselysine were compared per time point (expressed per 1 × 10^11^ bacterial cells (Fig. 4) and expressed per gram feces (Fig. S11). Identified outliers were excluded from further analyses (i.e. BF individual 12 at all time points, BF individual 20 at t = 2 h, FF individual 6 at t = 4 h). Comparing the two feeding modes at the different time points revealed that on group level, fecal samples from FF infants degraded on average significantly more fructoselysine compared to fecal samples from BF infants with an absolute difference of 14.9, 26.1, and 20.2 µmol/1 × 10^11^ bacterial cells at t = 2, t = 3, and t = 4 h, respectively (*P* < 0.05). Interindividual differences in the fructoselysine degradation activities were observed in both feeding modes as shown in Fig. 4, resulting in overlap in the range for the degradation capacity of the microbiota of the individuals from the two groups.

**Figure 5.**
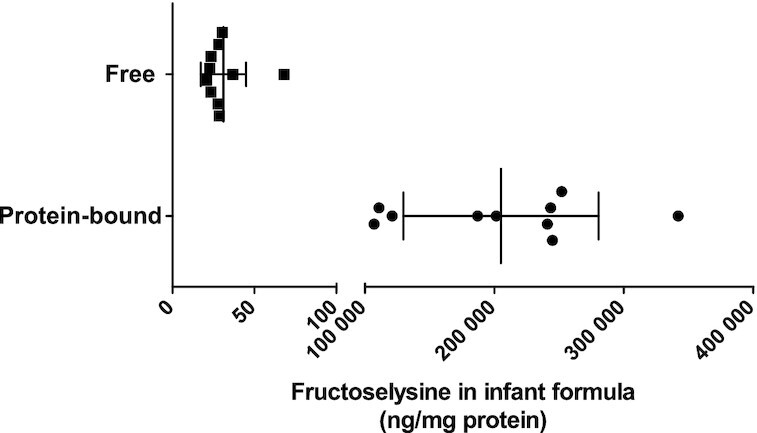
Amount of fructoselysine quantified in collected infant formula samples analyzed in both free and protein-bound form of fructoselysine per mg protein present in the infant formula. Data points represent average values of three repeated analyses of one infant formula sample.

To assess whether the interindividual differences in fructoselysine degradation activity of the FF infants could be explained by different levels of fructoselysine in their collected infant formula powder, protein-bound and free fructoselysine levels were quantified (Fig. 5). This revealed that in the formula powder the protein-bound proportion of fructoselysine (ranging from 107 to 342 µg/mg protein) was four orders of magnitude higher compared to the proportion of free fructoselysine (ranging from 21 to 68 ng/mg protein), and the quantified protein-bound levels were somewhat higher than previous reported data in literature (Fenaille et al. 2006). However, the interindividual differences in fructoselysine degradation activity of the FF infant fecal samples could not be explained by the protein-bound fructoselysine levels in the respective infant formula samples as the correlation between these parameters was not strong (R^2^ = 0.4; Fig. S12).

The high levels of fructoselysine in the infant formula were also reflected in the fecal fructoselysine excretion levels with an average of 11.6 µmol/g feces for the FF infants (Fig. S13), a result which is in line with the literature (Sillner et al. 2019). When extrapolating the *in vitro* degradation activities of the FF infants to the *in vivo* situation and comparing these to the estimated daily intake (EDI) as quantified based on the detected fructoselysine levels in infant formula, indeed only 28.6% of the amount of ingested fructoselysine originating from intake at the respective EDI, was estimated to be degraded by the obtained average degradation activity detected in the fecal samples from FF infants (Table S2). However, these results need to be interpreted with some caution as they are based on average values, estimations and assumptions (e.g. daily fecal weight).

To determine whether specific bacterial taxa present in the infant fecal samples were associated with fructoselysine degradation, a Spearman's rank correlation analysis was performed with taxa present in a relative abundance >0.1% in at least one of the individual fecal samples when transformed to absolute abundance glomerated at phylum (Fig. S14) or genus (Fig. S15) level. This analysis revealed that the phylum Bacteroidetes was shown to be significantly correlated with the amount of fructoselysine degraded (for BF: ρ = 0.85; adjusted *P*-value = 0.018) while no genus was significantly correlated.

## Discussion

In the present study, we show that the diet of infants affects the efficiency of the infant gut microbiota to degrade dietary derived fructoselysine. Fecal samples collected from FF infants showed a higher *in vitro* activity for microbial degradation of fructoselysine compared to the samples from BF infants. Search against a publicly available metagenome dataset revealed that sequences homologous to the ones of the functional genes involved in fructoselysine degradation are widely present in MAGs of both BF and FF infants, but with a higher prevalence in FF infants. The homologous sequences have a varying identity percentage to the query genes meaning functionality cannot be guaranteed, however, they were redundant amongst multiple bacterial species. Overall, the present study showed that fecal samples of infants exposed to different diets (i.e. breast milk and infant formula) resulting in different dietary fructoselysine exposures, differed in their microbial fructoselysine degradation activity. The microbial fructoselysine degradation activity was on average 6-fold higher for the pooled fecal samples from the FF infants compared to the pooled fecal samples from the BF infants.

Fructoselysine is a food process contaminant formed upon heating and processing of food products, and is highly present in infant formula due to the heating processes applied during production (Fenaille et al. 2006, Penndorf et al. 2007, Akıllıoğlu and Lund 2022). The high levels of fructoselysine in infant formula are in contrast to the low to no levels of fructoselysine in human breast milk (Martysiak-Zurowska and Stolyhwo 2007). This has been confirmed by the large differences in fecal excretion of fructoselysine by the two feeding groups as observed in the present study and reported in the literature (Sillner et al. 2019). This large difference in dietary fructoselysine exposure between BF and FF infants was accompanied by a higher microbial fructoselysine degradation activity of the fecal slurries from the FF infants, although the fecal slurries of the BF infants were also able to degrade fructoselysine. This implies that it is not possible to distinguish between BF and FF infants based on *in vitro* obtained fructoselysine degradation activities on an individual level, while on average group level it is. This is in line with the metagenomic dataset analysis as we observed sequences homologous to the relevant genes in question (frlD/yhfQ) also in the BF infant fecal samples, although with a lower corresponding prevalence as compared to the FF infant fecal samples. However, our observations are not completely in agreement with a previous study where two inoculates of BF infant fecal samples were unable to degrade fructoselysine, while two FF infants were able to do so (Bui et al. 2020).

In the present study the bacterial composition of the collected fecal samples did not differ significantly between the BF and FF infants, and the feeding mode was for this group of infants not the main driver determining the variance in microbiota composition. Possibly a higher resolution might be needed to characterize differences in the bacterial composition between the two groups as also indicated upon the metagenomic dataset analysis. Similar to these observations, the interindividual differences in microbial fructoselysine degradation activity of both the BF and FF infant fecal samples could not be explained by differential abundance of specific bacterial genera, indicating that higher resolution bacterial composition data is needed (i.e. up to species or strain level), that identical bacteria might be capable of multiple functionalities depending on the compounds they ferment and/or that multiple bacteria could be potentially involved. This is in line with the results of the metagenomic data set analysis focusing on the frlD/yhfQ genes because of the following. First of all, the homologous sequences were widely present in different genera that are part of different phyla, both in the fecal data of BF and FF infants of the metagenomic data set. In addition, at species level the sequences were found in different species that were part of the same overarching genus. Together this indicates that differences in potential fructoselysine degradation activities can go down to at least species level. In line with the higher *in vitro* fructoselysine degradation activity of the fecal samples from the FF infants compared to the BF infant fecal samples, the potential genes involved in fructoselysine degradation (i.e. frlD/yhfQ) were found in a higher prevalence in feces from FF infants compared to that from BF infants. A previous study found genes involved in fructoselysine metabolism in metagenomes of most of the FF infants (56%) and only in some BF infants (10%) (Bui et al. 2020). However, in this previous study the complete fructoselysine metabolism pathway was considered (Bui et al. 2015) while the analysis of the present study focused on the first step in the fructoselysine degradation.

The results combined show that the potential fructoselysine degradation function is redundant in the metagenomes of both BF and FF infant fecal samples, but that fecal samples collected from FF infants, which have been exposed to fructoselysine through their diet, showed a higher fructoselysine degradation activity *in vitro* compared to fecal samples from BF infants. This suggests that direct dietary exposure might be responsible for an increase in activity of the microbes involved, which might also -at least partly- explain interindividual differences in fructoselysine degradation observed with adult human fecal samples (van Dongen et al. 2021b).

The fructoselysine degradation activity of the FF infants (average 3.42 µmol/h/g feces) was comparable to the average of 16 individual adults (3.4 µmol/h/g feces) as shown in our previous study (van Dongen et al. 2021b). However, based on these degradation rates and the daily defecation rates, it was calculated that, due to the high fructoselysine intake by FF infants, only 28.6% of the estimated daily fructoselysine intake was expected to be microbially degraded. This estimation of incomplete fructoselysine degradation is supported by the quantified levels of fructoselysine still being present in the collected fecal samples of the FF infants. This incomplete fructoselysine degradation implies a direct exposure to fructoselysine in the intestine of the infants. Whether this can affect (local intestinal) human health remains to be further investigated, as well as whether subsequent fructoselysine exposure affects microbial composition itself, which will also depend on the characteristics of the compound itself as well as the dose. In the present study the microbial composition of the collected fecal samples did not differ between the BF and FF infants, while diets high in AGEs have previously been reported to affect fecal microbiota composition (Seiquer et al. 2014, van Dongen et al. 2021a). However, it remains unclear whether those reported alterations can be exclusively attributed to the different AGE levels in the provided diets, and it is important to note that the two different feeding modes studied in the present study (i.e. BF and FF) are not differing solely in their fructoselysine content.

In summary, a metagenomic data set analysis revealed that particular genes involved in microbial fructoselysine degradation were present in fecal samples from both BF and FF infants, although with a higher prevalence in the fecal samples from the FF infants. Additional collected fecal samples from FF infants appeared to show a higher fructoselysine degradation activity *in vitro* compared to the collected fecal samples from BF infants, an observation that can likely be ascribed to relatively higher dietary fructoselysine exposure of the gut microbiota from the FF infants. This suggests that, at least for the AGE precursor fructoselysine, the (infant) gut microbiota can adapt towards exposures via the environment through e.g. the diet.

## Supplementary Material

fiac145_Supplemental_File
