# Supplementary material for: Differences in gut microbial fructoselysine degradation activity between breast-fed and formula-fed infants
Source: FEMS Microbiol Ecol. 2022 Nov 28;99(1):fiac145. doi: 10.1093/femsec/fiac145 (PMC9749803; doi:10.1093/femsec/fiac145)
Supplement: fiac145_Supplemental_File [file fiac145_supplemental_file.docx]

**Differences in gut microbial fructoselysine degradation activity between breast-fed and formula-fed infants**

Katja C.W. van Dongen^1^, Athanasia Ioannou^2^, Sebastiaan Wesseling^1^, Karsten Beekmann^3^, Clara Belzer^2*^

1. Division of Toxicology, Wageningen University and Research, the Netherlands
2. Laboratory of Microbiology, Wageningen University and Research, the Netherlands
3. Wageningen Food Safety Research, Wageningen University and Research, the Netherlands

*Corresponding author:

Clara Belzer

Laboratory of Microbiology

Stippeneng 4

6708 WE Wageningen

the Netherlands

clara.belzer@wur.nl

+31317482795

## Supplementary materials

**Supplementary tables**

***Table S1*** *Sequences used for blastp against the created MAGs (metagenome assembled genomes) database*

| Abbreviation | Origin | Protein name | GenBank Accession No. | Reference |
| --- | --- | --- | --- | --- |
| frlD | *Escherichia coli* str. K-12 substr. MG1655 | Fructoselysine kinase | NP_417833.1 | (Wiame *et al.* 2002) |
| frlD | *Bacillus subtilis* subsp. Subtilis str. 168 | Fructoselysine kinase | NP_391137.1 | (Wiame *et al.* 2004) |
| yhfQ | *Intestinimonas butyriciproducens* str. AF211 | Fructoselysine kinase | ALP93343.1 | (Bui *et al.* 2015) |
| frlB | *Bacillus subtilis* subsp. Subtilis str. 168 | Fructosamine-6-phosphate deglycase | NP_391141.1 | (Wiame *et al.* 2004) |
| frlB | *Escherichia coli* str. K-12 substr. MG1655 | Fructosamine-6-phosphate deglycase | NP_417830.4 | (Wiame *et al.* 2002) |
| yhfN | *Intestinimonas butyriciproducens* str. AF211 | Fructosamine-6-phosphate deglycase | ALP93345.1 | (Bui *et al.* 2015) |

***Table S2*** *Calculation of scaling the in vitro determined degradation parameters to the in vivo situation*

|  | Average fructoselysine in tested formula samples (mg/g protein) | Daily intake (mg per 24h)^a^ | Daily intake (µmol per 24h)^b^ | Average in vitro degradation capacities (µmol/g feces/h)^c^ | In vivo degradation capacity in 24h (µmol)^d^ | % Of daily intake can be degraded in 24h |
| --- | --- | --- | --- | --- | --- | --- |
| Formula-fed infants | 205.1 | 2744.8 | 8903.0 | 3.42 | 2544.5 | 28.6 |

^a^ Assuming 9.7% protein content per gram infant formula powder, 6 scoops each 4.6 g per bottle, 5 bottles a day. Based on ‘Nutrilon Zuigelingenvoeding 1’ online guidelines.

^b^ Molecular weight is 308.3 g/mol.

^c^ Degradation capacities were experimentally obtained in the present study and represent the averaged individual degradation rates per feeding group which were assessed by averaging the three measured incubation time points per individual fecal sample of all tested fecal samples per feeding group

^d^ Transformed by assuming a daily fecal mass of 31g over 24h (Lemoh J.N. and Brooke O.G. 1978; Gustin *et al.* 2018).


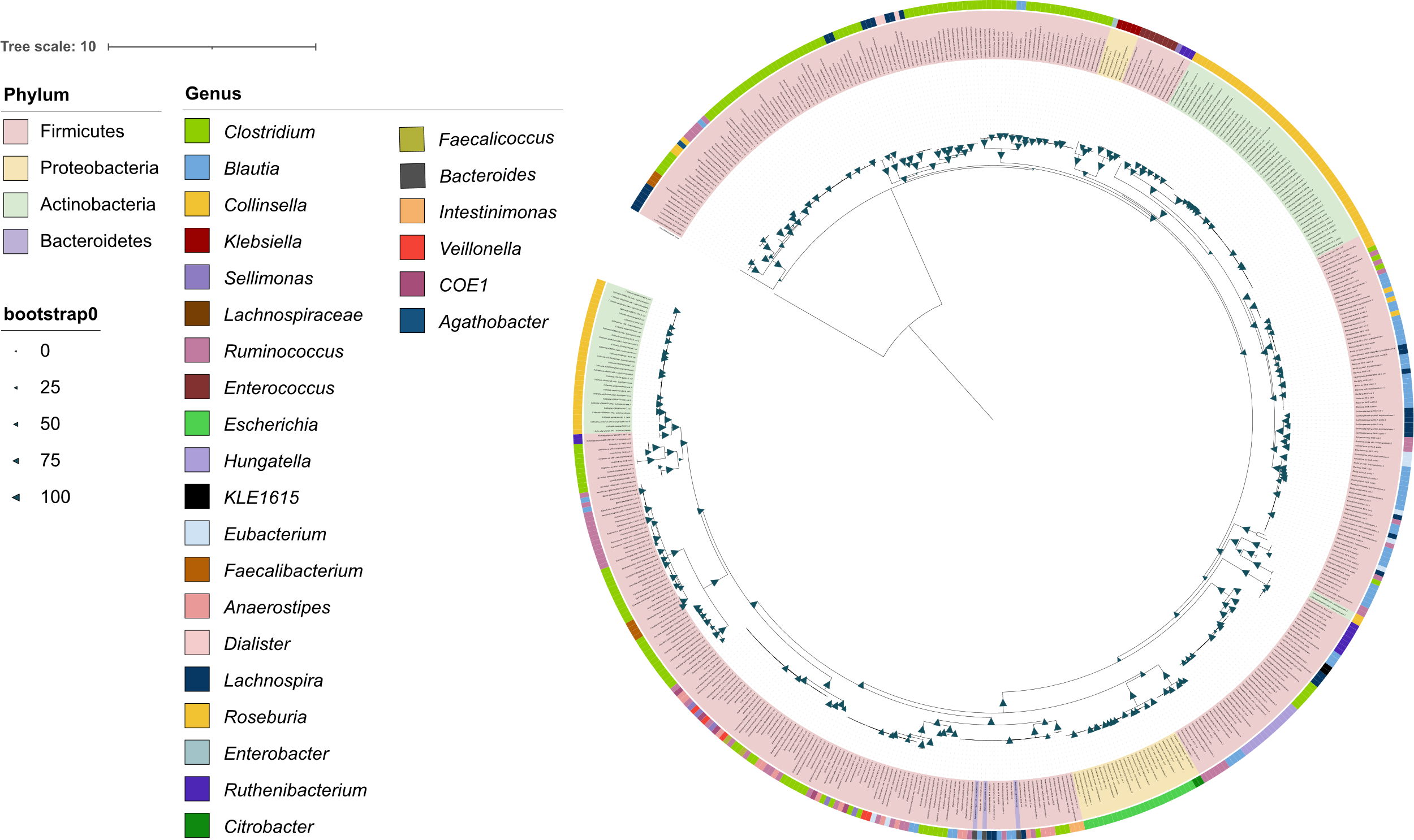
**Supplementary figures**

**Figure S1** iTol phylogenetic tree of the genes frlD and yhfQ, responsible for the degradation of fructoselysine into fructoselysine-6-phosphate. Bacterial taxa with frlD/yhfQ genes at phylum and genus level are highlighted in assigned colors. Species are mentioned at tree edges.


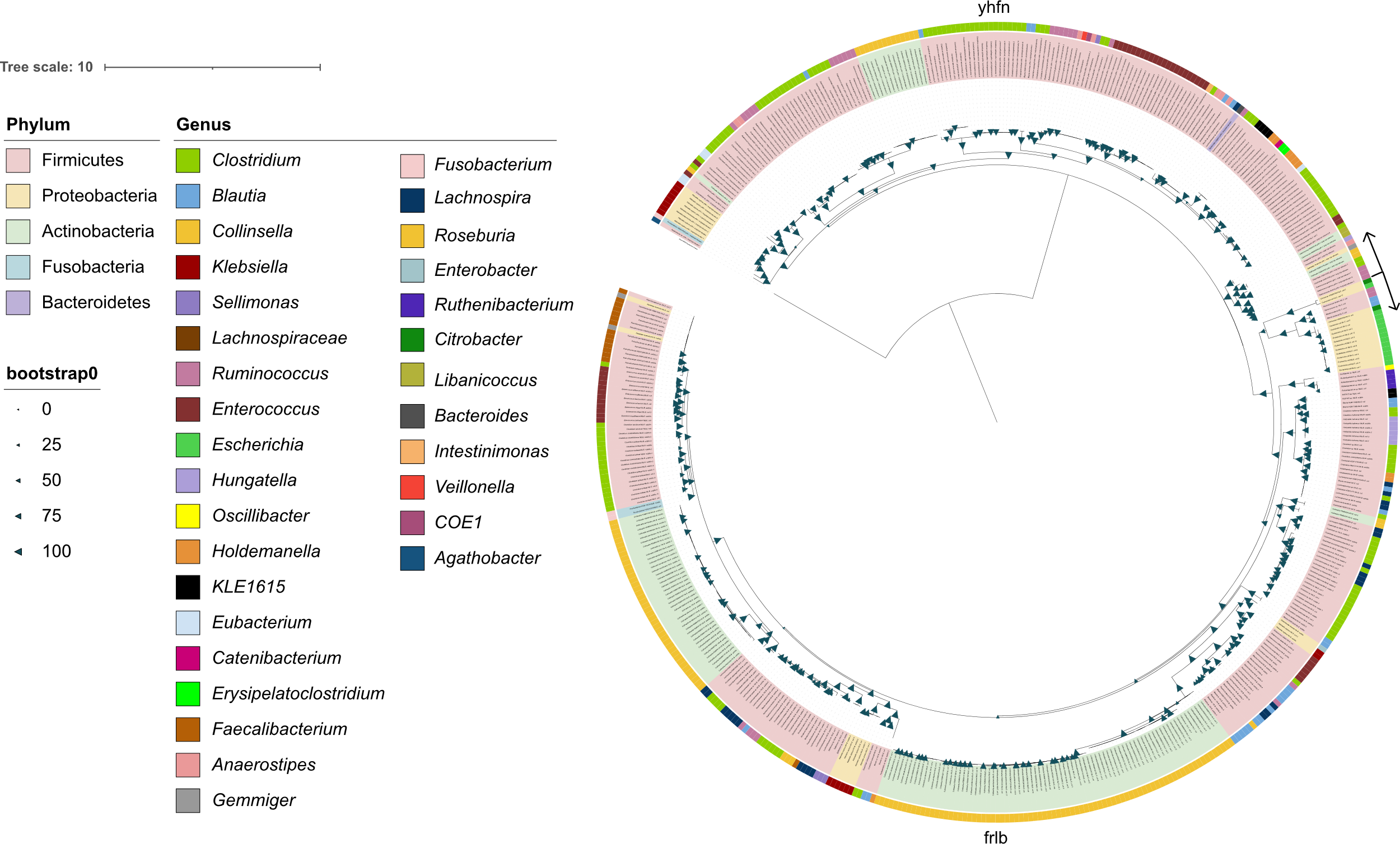
**Figure S2** iTol phylogenetic tree of the genes frlB and yhfN, responsible for further metabolism of fructoselysine-6-phosphate. Bacterial taxa with frlB/yhfN genes at phylum and genus level are highlighted in assigned colors. Species are mentioned at tree edges.

**
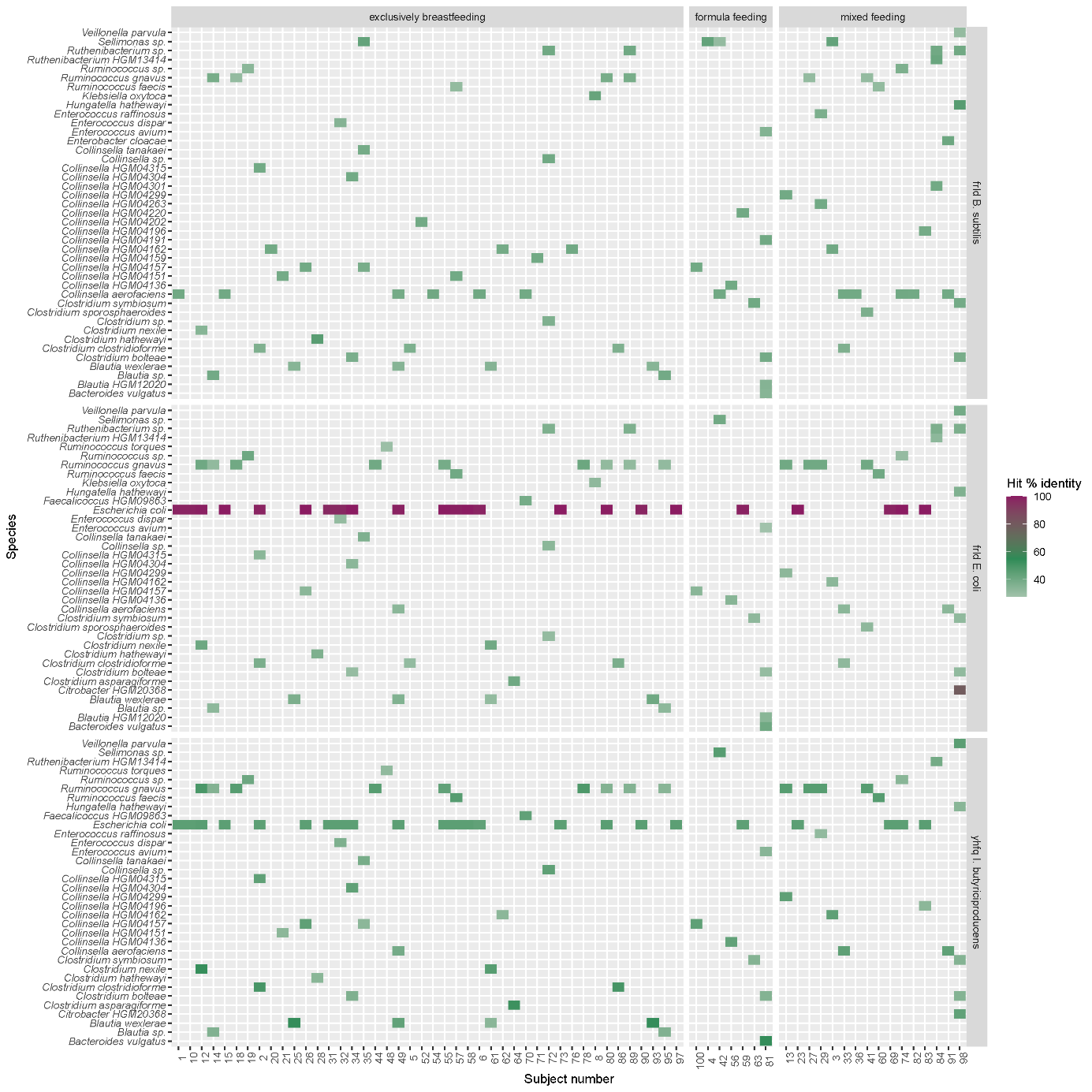
**

**Figure S3** Heatmap of hits of the query genes against the included metagenome assembled genomes (MAGs), for infants aged 4 months. The mean percentage identity of each hit per query and species per subject is depicted. The taxa identity mentioned in the figure was the lowest identified taxonomic hierarchical level possible.

**Figure S4** PCoA plot of Bay-Curtis beta diversity dissimilarities of 20 individual fecal samples of exclusively breast-fed (BF; red circles) or formula-fed (FF; blue triangles) infants.


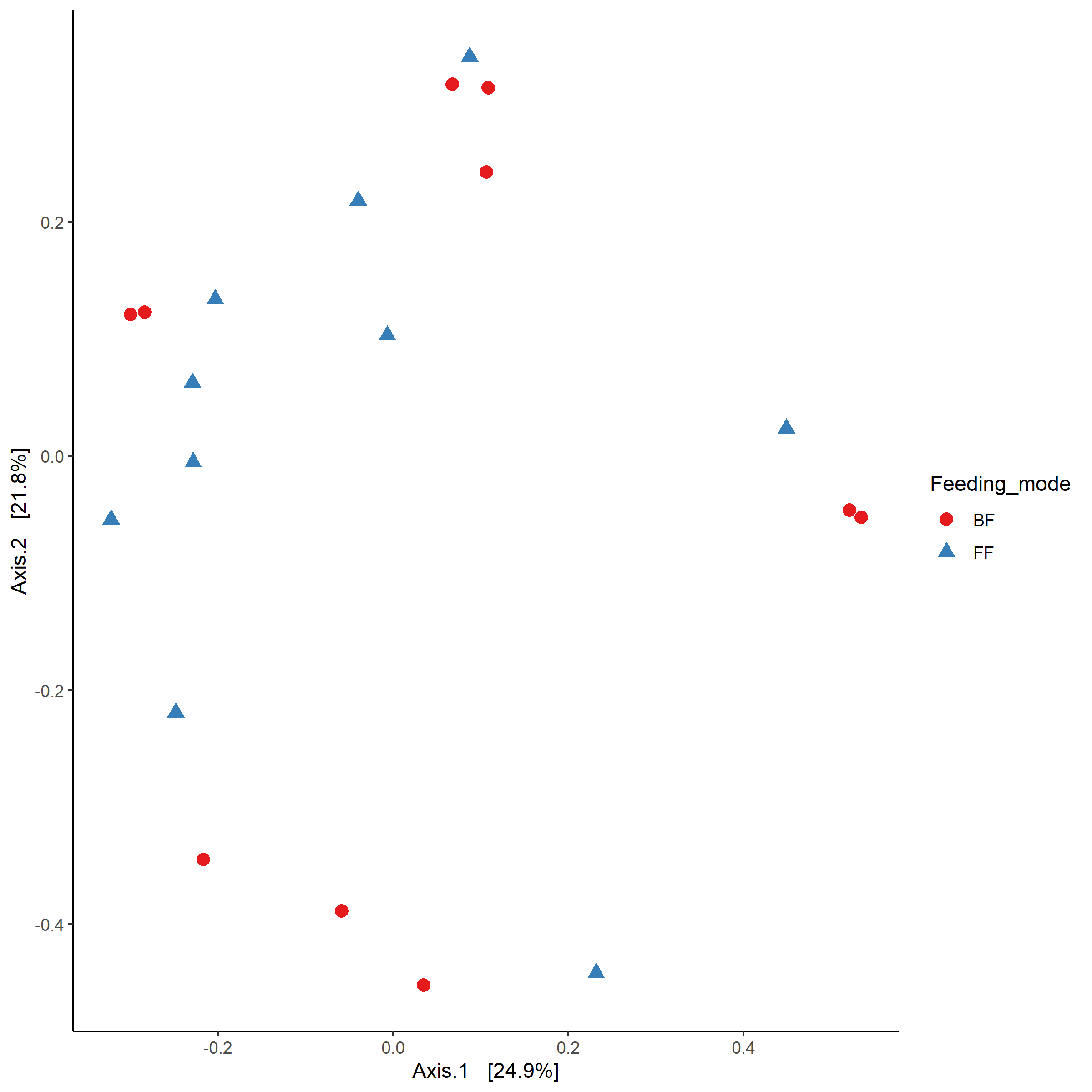


***Figure S5*** *Absolute abundance of microbial taxa, assessed with 16S rRNA amplicon sequencing and qPCR, present in fecal samples either exclusively breast-fed (BF) or formula-fed (FF) infants. The top 10 taxa present at phylum (panel A) and genus (panel B) level are shown. Y-axis labels indicate feeding mode and subject number.*


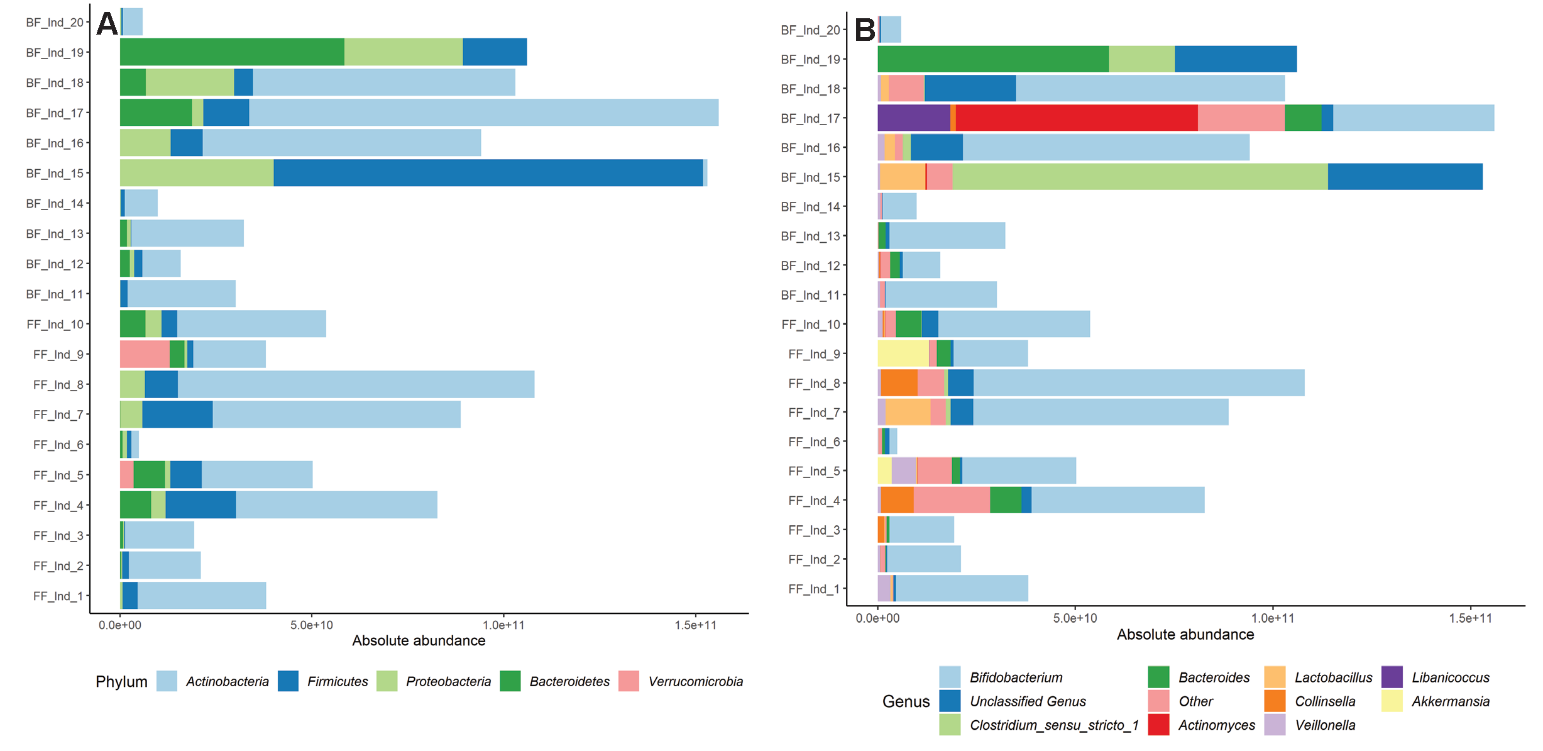


**Figure S6** Relative abundance of microbial taxa, assessed with 16S rRNA amplicon sequencing and qPCR, present in infant fecal samples either exclusively breast-fed (BF) or formula-fed (FF). The top 10 taxa present at phylum (panel A) and genus (panel B) level are shown. Y-axis labels consist of feeding mode and subject number.


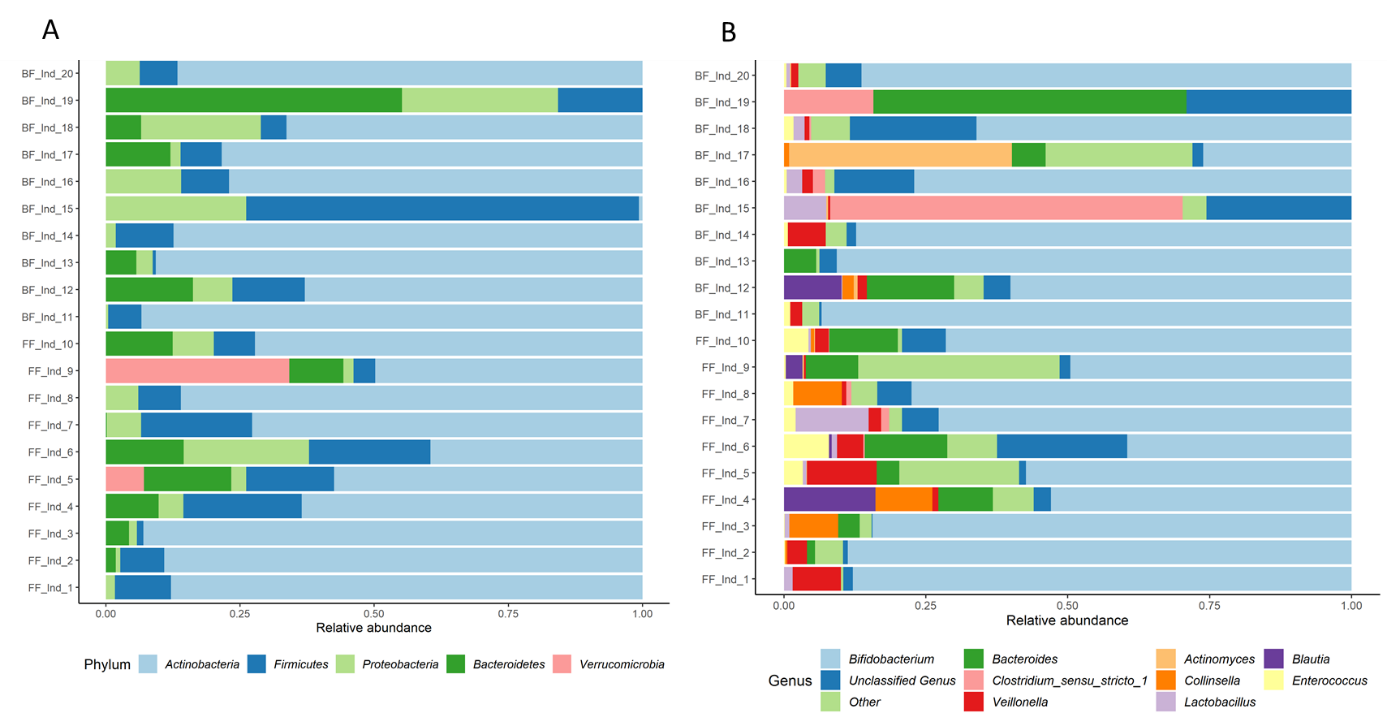

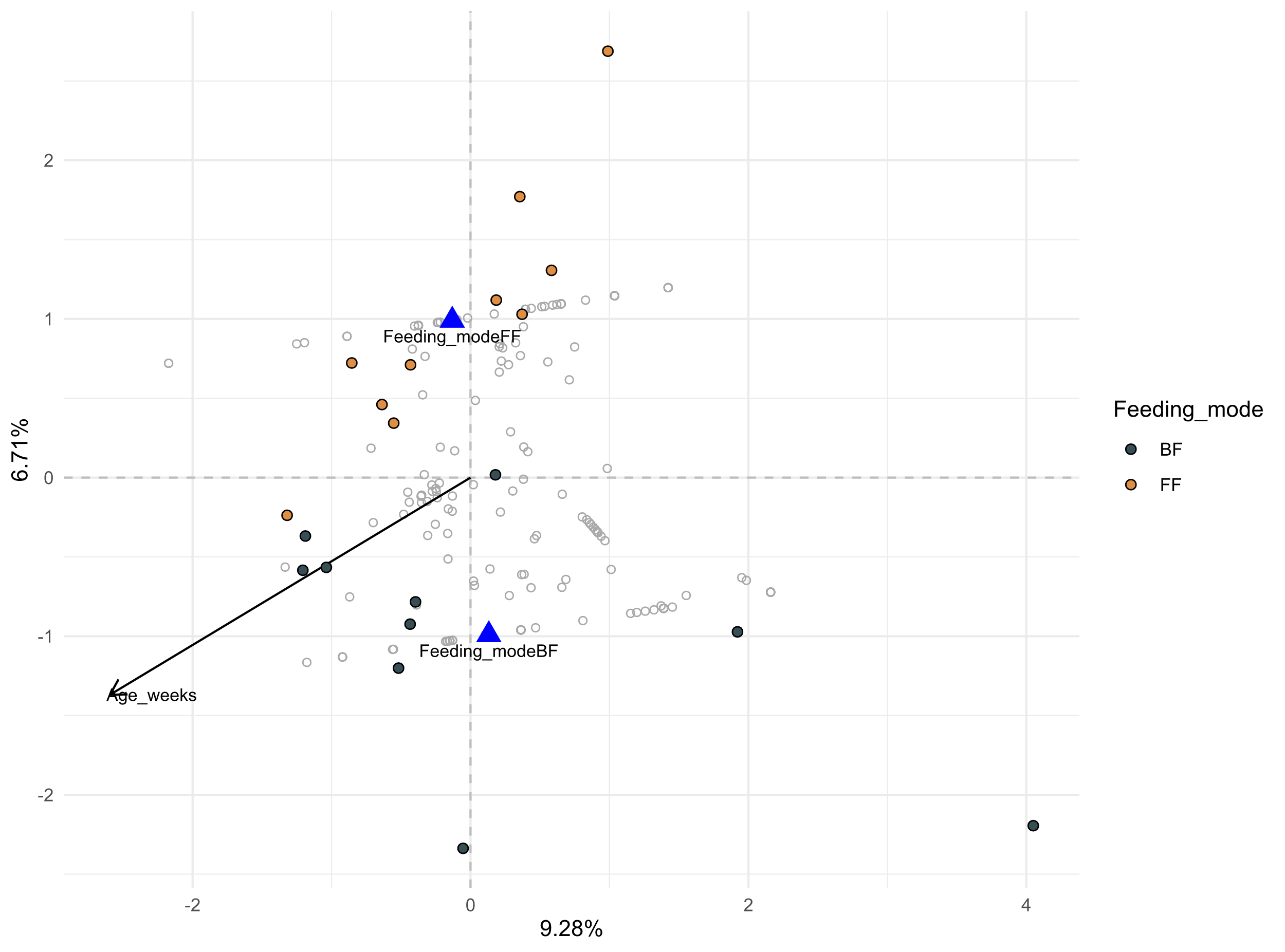


CCA1

CCA2

**Figure S7** Canonical correspondence analysis (CCA). Grey open circles represent bacterial taxa; closed circles represent the infant fecal samples (black breast-fed BF; orange formula-fed FF); blue triangles represent the centroids of the FF and BF infant fecal samples. Axis percentages represent the percentage of variation explained only by the constraining variables (i.e. feeding mode and age).

**Figure S8** Total bacterial cell load per gram fecal sample, as collected per individual for both feeding groups (FF formula-fed; BF breast-fed). Total bacterial cell load was determined by qPCR and data represent the average ± SD of three technical replicates.

**Figure S9** Amount of degraded fructoselysine upon anaerobic incubation of fructoselysine (final substrate concentration 430 µM) with pooled fecal slurries (final fecal concentration 0.0125 g/mL) of infants exclusively breast-fed (BF) or formula-fed (FF), containing 10 infant fecal samples per feeding mode. Data points represent the average ± SD of three independent repeated experiments. Differences between the BF and FF results were assessed for statistical significance per time point by a 2-way ANOVA followed by Bonferroni post-hoc test: * p-value <0.05; ** p-value < 0.01; *** p-value < 0.001; **** p-value < 0.0001.


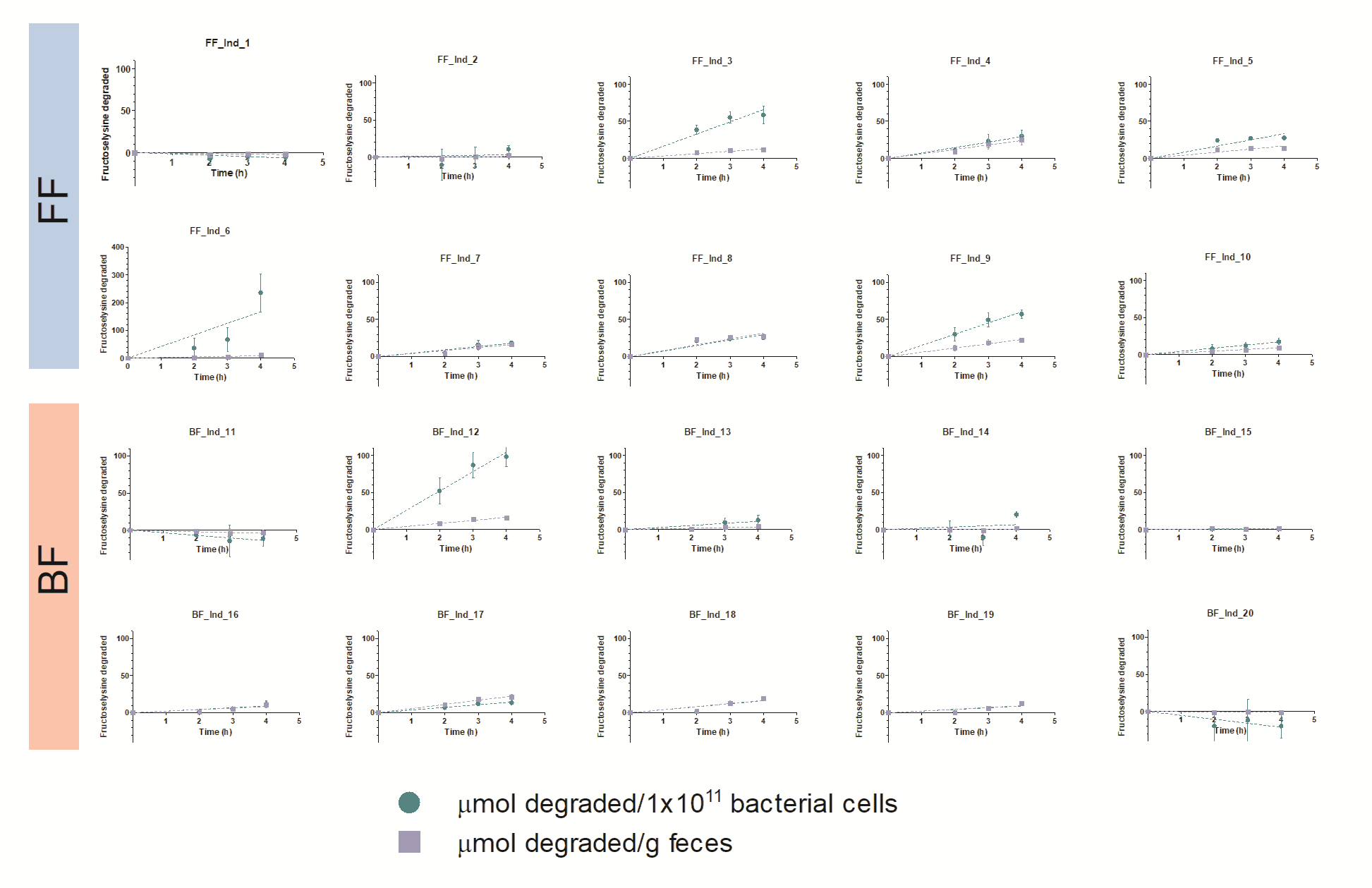


**Figure S10** Amount of degraded fructoselysine upon anaerobic incubation of fructoselysine (final substrate concentration 430 µM) with individual fecal slurries (final fecal concentration 0.0125 g/mL) of infants exclusively breast-fed (BF) or formula-fed (FF), containing 10 infant fecal samples per feeding mode. Data points represent the average ± SD of three independent repeated experiments. Dotted lines represent the slope.

**Figure S11** Amount of degraded fructoselysine by individual fecal samples from exclusively breast-fed (BF) or formula-fed (FF) infants quantified at each anaerobic incubation time point (i.e. 2, 3 or 4 hours). Scatter dots indicate average values of three independent experiments for each individual fecal sample. Center bars indicate average values while whiskers indicate the standard deviation. Whether the values of the two feeding modes were significantly different for each respective incubation time was evaluated with an unpaired t-test. Identified outliers were indicated with an open symbol and excluded for further analyses.

**Figure S12** Correlation of amount of degraded fructoselysine of formula-fed infants and protein-bound fructoselysine levels in their infant formula. The rate of fructoselysine degradation represents the average degradation rate per individual of the three measured incubation time points.


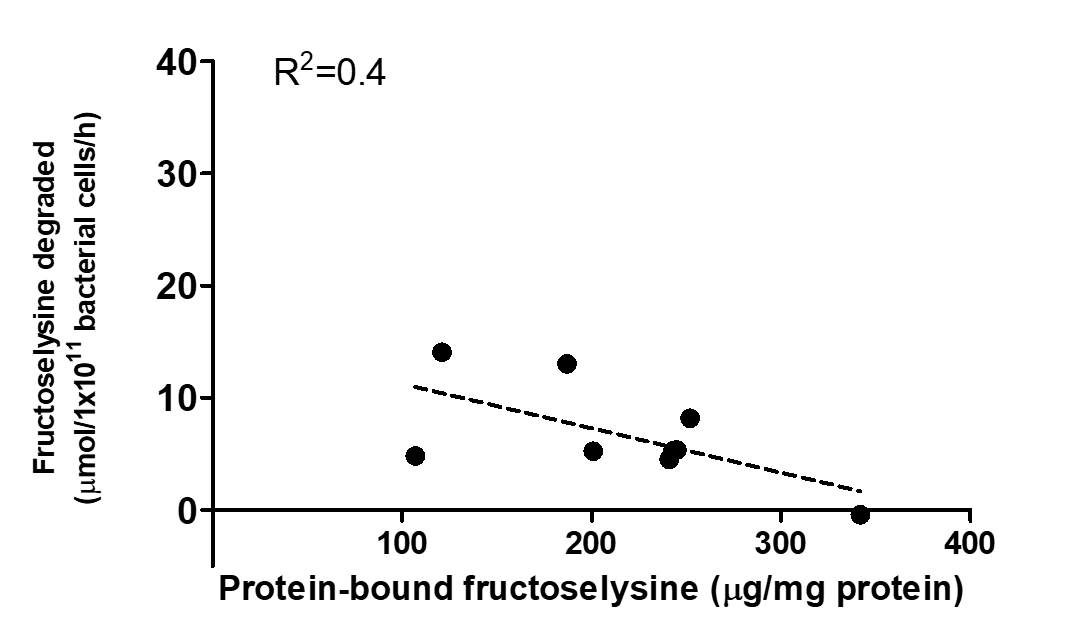


**Figure S13** Fructoselysine fecal excretion in exclusively formula-fed (FF) or breast-fed (BF) infant fecal samples.


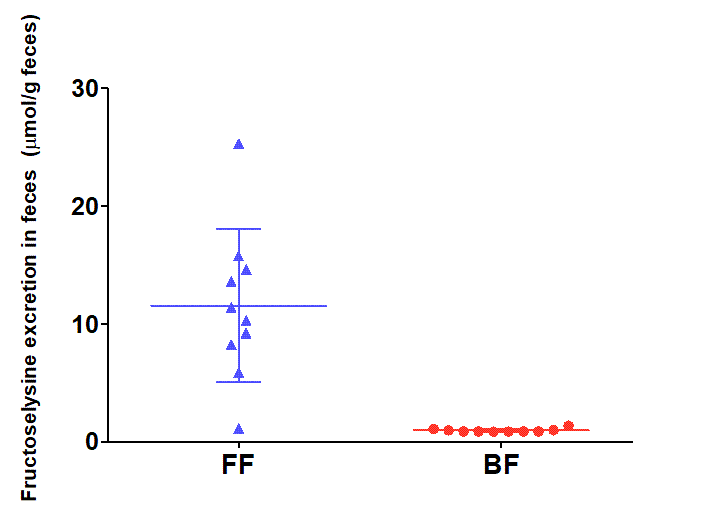


**Figure S14** Spearman’s rank correlation analysis of bacterial taxa at phylum level with the amount of degraded fructoselysine per gram feces per hour. This degradation rate represents the average degradation rate of the three measured incubation time points per individual fecal sample. Bacterial taxa present with a relative abundance >0.1% in one of the individual fecal samples were included and transformed into absolute abundance (using quantified total bacterial cell load by qPCR). P-values were adjusted for multiple testing by FDR and indicated as follows: * P-value < 0.05.

*


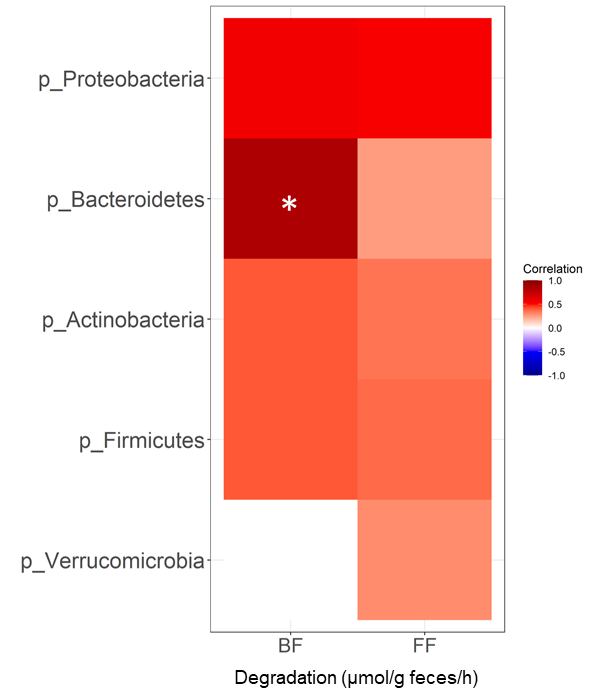

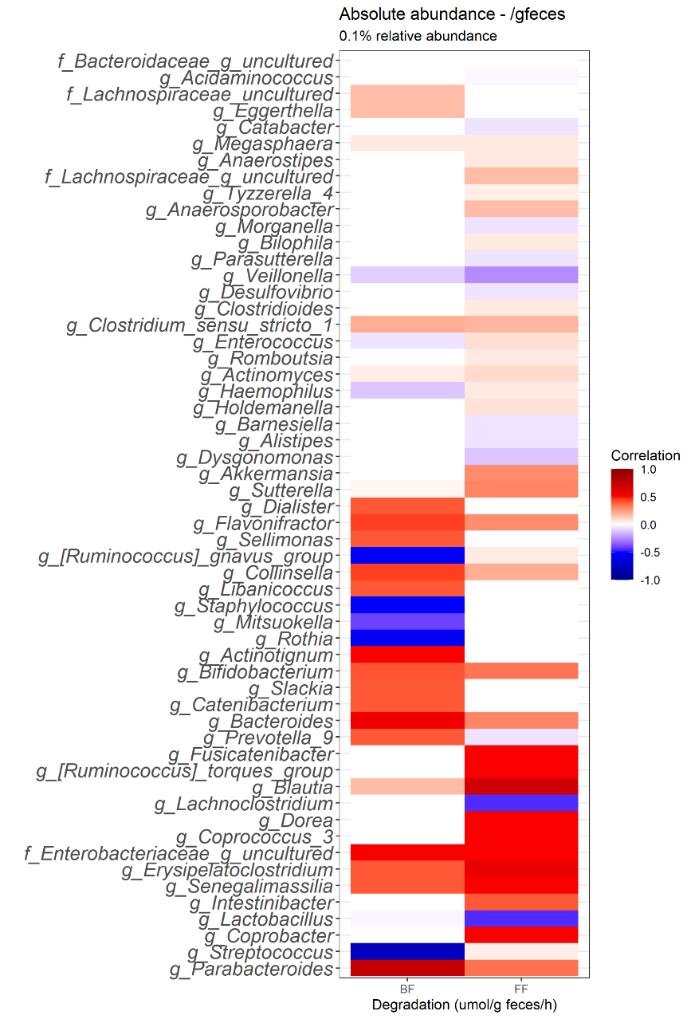


**Figure S15** Spearman’s rank correlation analysis of bacterial taxa at genus level with the amount of degraded fructoselysine per gram feces per hour. This degradation rate represents the average degradation rate of the three measured incubation time points per individual fecal sample. Bacterial genera present with a relative abundance >0.1% in one of the individual fecal samples were included and transformed into absolute abundance (using quantified total bacterial cell load by qPCR). P-values were adjusted for multiple testing by FDR.
